# Using a Harmonic Scalpel “Drilling and Clamping” Method to Implement Zero Ischemic Robotic-assisted Partial Nephrectomy: An Observation Case Report Study

**DOI:** 10.1097/MD.0000000000002349

**Published:** 2016-01-22

**Authors:** Chen-Pang Hou, Yu-Hsiang Lin, Yu-Chao Hsu, Chien-Lun Chen, Phei-Lang Chang, Ke-Hung Tsui

**Affiliations:** From the Department of Urology, Chang Gung Memorial Hospital at Linkou (K-HT, P-LC); School of Medicine, Chang Gung University (K-H T, P-LC, C-PH, Y-HL, Y-CH, C-LC); Kwei-Shan, Tao-Yuan, Taiwan.

## Abstract

Robot-assisted partial nephrectomy (RAPN) has gradually become a popular minimally invasive nephron-sparing surgical option for small renal tumors. Ischemic injury should be minimized because it impacts renal function outcomes following partial nephrectomy. Herein, the authors detail the technique and present initial perioperative outcomes of our novel harmonic scalpel “drilling and clamping” method to implement zero-ischemic RAPN. The authors prospectively collected baseline and perioperative data of patients who underwent zero ischemic RAPN performed by our harmonic scalpel “drilling and clamping” method. From April 2012 to December 2014, a total of 19 consecutive zero ischemic RAPN procedures were performed by a single surgeon. For 18 of the 19 patients, RAPN using our harmonic scalpel “Drilling and Clamping” method was successfully completed without the need for hilar clamping. The median tumor size was 3.4 cm (range: 1.8–6.2); operative time was 3.2 hours (range: 1.9–4.5); blood loss was 100 mL (range: 30–950); and postoperative hospital stay was 4 days (3–26). One patient required intraoperative blood transfusion. Two patients had intra or postoperative complications: 1 was converted to traditional laparotomy because of massive bleeding, whereas another had postoperative stress ulcer. Pathology confirmed renal cell carcinoma in 13 patients (63.2%), angiomyolipoma in 6 patients: (31.5%), and oncocytoma in 1 patient (5.3%). Mean pre- and postoperative serum creatinine (0.82 mg/dL and 0.85 mg/dL, respectively), estimated glomerular filtration rate (84.12 and 82.18, respectively), and hemoglobin (13.27 g/dL and 12.71 g/dL, respectively) were comparable. The authors present a novel zero-ischemic technique for RAPN. They believe that this technique is feasible and reproducible.

## INTRODUCTION

Compared with radical nephrectomy, nephron-sparing surgery for smaller-sized tumors provides equivalent oncologic and superior functional outcomes.^1,2^ Therefore, partial nephrectomy has been the standard surgical treatment for clinical T1 renal tumors (<7 cm).^3^ The clinical outcomes of laparoscopic partial nephrectomy has been well discussed in the literature.^4–7^ Because robot-assisted partial nephrectomy (RAPN) was introduced in 2004, it has been an alternative to traditional laparoscopic partial nephrectomy.^8,9^ Typical partial nephrectomy techniques require hilar clamping to reduce the blood loss during tumor excision, and it was believed that renal injury from a limited warm ischemia time of <20 to 30 minutes is transient and reversible.^10^ The study of Thompson et al, however, revealed that longer warm ischemia time is associated with short- and long-term renal consequences, and every minute counts when the renal hilum is clamped.^11^ The negative impact of renal ischemia could be even higher in patients with underlying renal dysfunction, preexisting comorbidities, or advancing age.^12^ Therefore, how to reduce the ischemic injury during partial nephrectomy is crucial. Recently, many studies regarding zero-ischemic RAPN have been published in the literature.^13–15^ Herein, we detail the technique and present initial perioperative outcomes of our novel harmonic scalpel “drilling and clamping” method to implement zero-ischemic RAPN.

## METHODS

### Study Population

Data were collected prospectively and entered into an institutional review board-approved data base of Chang-Gung Memorial Hospital. Patients with single, exophytic, clinical T1 tumor who were deemed to be candidates for RAPN were included in the study. Patients with endophytic or hilar tumor, high operative risks with severe, preexisting cerebrovascular, cardiopulmonary, or hepatorenal dysfunction were excluded from the study. From April 2012 to December 2014, 19 consecutive patients consented to “zero-ischemic” RAPN.

### Preoperative Work-up and Data Collection

**FIGURE 1:**
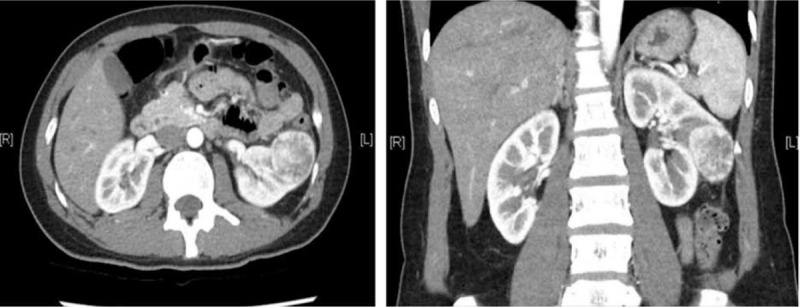
Preoperative CT scans showing the renal tumor to be treated with zero ischemia robot-assisted partial nephrectomy: A 51-year-old female patient with a left 3.6 cm exophytic midpole renal tumor.

The patients underwent a detailed medical history assessment, physical examination, and routine laboratory tests. Serum creatinine was recorded preoperatively and at 1 month after operation, while hemoglobin (Hb) was recorded preoperatively and at discharge day for this study. The modification of diet in renal disease (MDRD) was used for calculating the estimated glomerular filtration rate (eGFR). Before the operations were performed, all patients received three-dimensional abdominal computed tomography with 2 to 3-mm cuts to delineate the tumor location, depth, and the distance to the collecting system (Figure 1). Percentage change in serum creatinine, eGFR, and Hb were determined by calculating the difference between the preoperative and postoperative data. Bowel preparations were not necessary before the day of surgery.

### Preparations:

**FIGURE 2:**
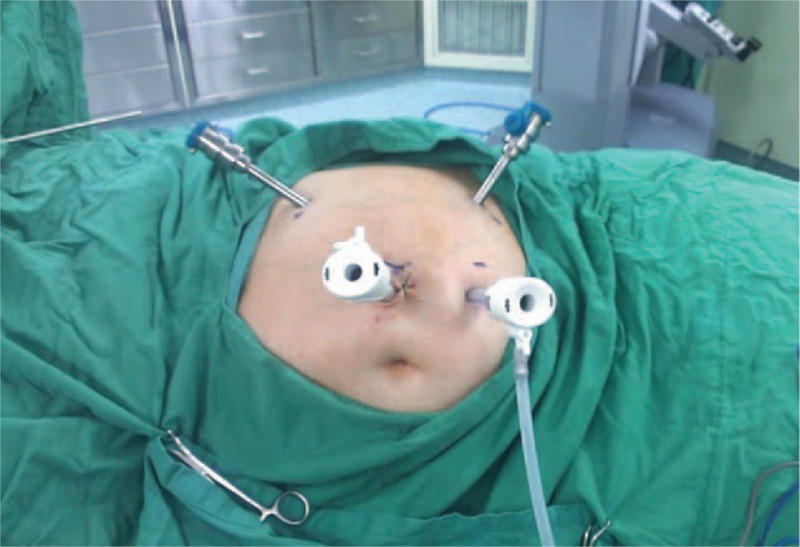
Patient position: The patient was secured to the operation table, with a modified lateral position. The table was positioned at 20 to 30° contralateral tilt and 30° angle relative to the robot. Illustration of trocar placement: a 12 mm camera port, 2 8 mm robotic working ports, and a 12 mm assistant port.

**FIGURE 3:**
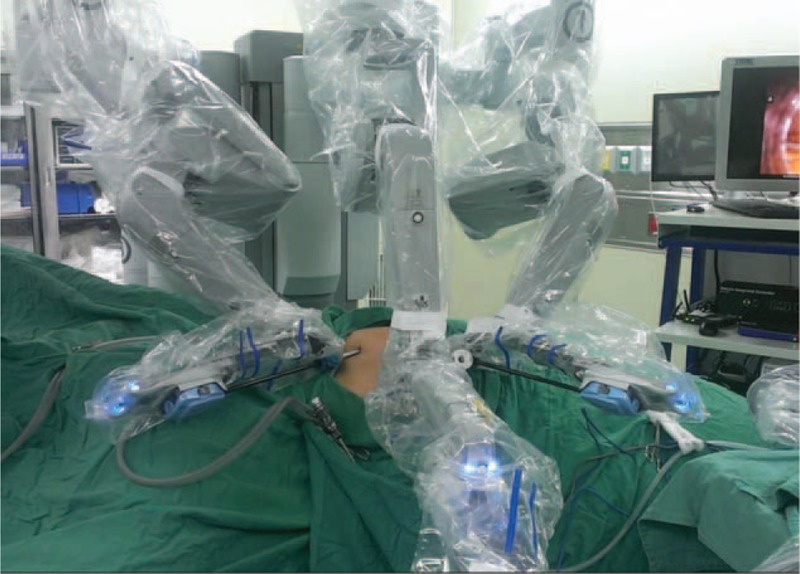
Docking the robot: The robot was docked over the ipsilateral shoulder at a 30° angle from the patient's spine.

1. Patient position: The patients were placed in a modified lateral position (30 degree tilt). The pressure points were carefully padded with foam pads and pillows. The table was positioned at 20 to 30° contralateral tilt (Figure 2).
2. Trocar position: All the operations were performed transperitoneally. After pneumoperitoneum was established, the camera port, using a 12 mm trocar, was achieved 10 cm lateral and 3 cm superior to the umbilicus. One robotic working port (8 mm trocar) was placed 3 cm medial to the ipsilateral anterior superior iliac spine. Another working port (8 mm trocar) was placed below the costal margin in the mid-clavicular line. A 12 mm trocar was used as the assistant port, located at 5 cm inferior to the camera port (Figure 2). The port placement would be modified to a more lateral position if the case was a high body mass index patient.
3. Docking the robot: The robot was docked over the ipsilateral shoulder at a 30° angle from the patient's spine (Figure 3).

### Surgical Procedures:

**FIGURE 4:**
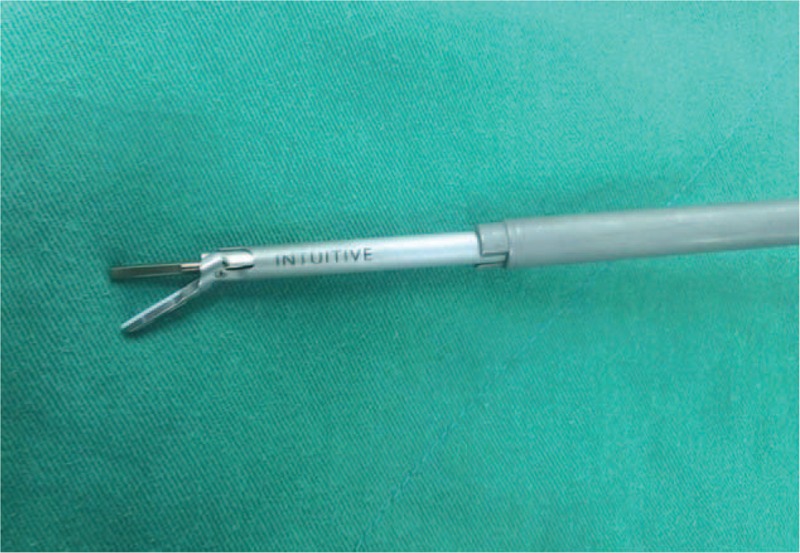
Harmonic scalpel (Intuitive Surgical, Sunnyvale, CA) used for zero ischemia robot-assisted partial nephrectomy.

**FIGURE 5:**
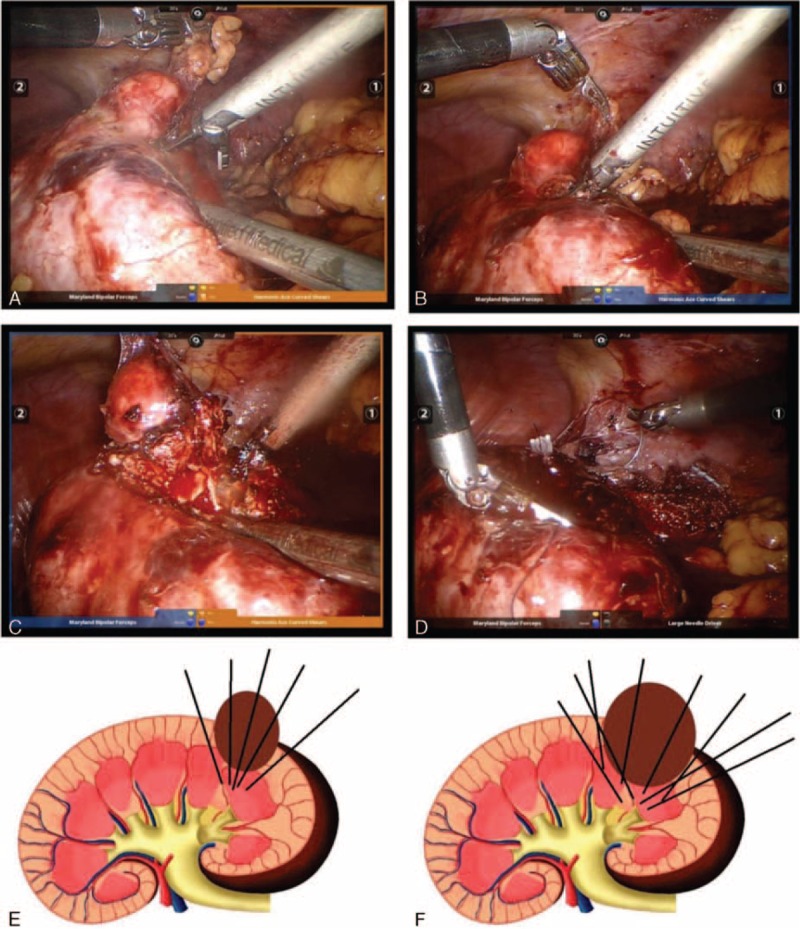
A, Drill holes by robotic harmonic scalpel around the tumor, with a 5 mm distance from the tumor margin. The distance between each hole was approximately 3 mm. B, Insert the scalpel blades into the created holes, clamp, and cut. C, Repeat the drilling and clamping method around the tumor outline to excise the mass. E, The drilling angle should be beveled tangent to the surface arc of the tumor. F, For a larger tumor, the technique can be performed again on the second layer until the whole mass is completely resected.

1. Zero-degree camera lens was used during the whole procedure.
2. Once the colon was taken down, the Gerota fascia was opened, and the ureter as well as the kidney low pole was lifted off the psoas muscle. The kidney was mobilized for identifying the tumor. For upper pole or posterior aspect tumors, the entire kidney should be mobilized from the abdominal side wall and diaphragm as much as possible. Intraoperative ultrasonography was used to identify the tumor location. Identification of the hilum and vessels that might require clamping was not necessary in our surgical technique.
3. We used the blunt blade of robotic Harmonic scalpel (Intuitive Surgical, Sunnyvale, CA) (Figure 4) to drill holes around the tumor, with a 5 mm distance from the tumor margin. The distance between each hole was approximately 3 mm (Figure 5A). The drilling angle should be beveled tangent to the surface arc of the tumor (Figure 5E). We inserted the scalpel into the created holes and clamped. The kidney tissue was cut at power level 5 and 55,500 Hz vibrating frequency (Figure 5B). The “drilling and clamping” method was repeated around the tumor outline to excise the mass (Figure 5C). As for larger tumors, the technique could be performed again on the second layer until the whole mass was completely resected (Figure 5F).
4. A 3–0 self-locking, absorbable polyglyconate suture (V-lock 90TM; Covidien Inc) was used to close the large sinuses, end arteries, and collecting system at the resection base. After the bleeding was controlled, 3–0 Vicryl sutures were placed across the defect to close the parenchyma using a sliding renorraphy technique with large Hem-o-lock clips (Teleflex Medical) (Figure 5E).^16^
5. FLOSEAL Hemostatic Matrix (Baxter Inc, CA) and Surgicel (Ethicon Inc, Somerville, NJ) were applied to the resection bed before the whole procedure terminated.

### Postoperative Care

The patients received clear liquid diet on the postoperative day 1 and would advance to regular diet as they tolerate it. The Jackson–Pratt drain placed during the operation was removed if the drainage of excess fluid from the surgical area was less than 50 cc per day. The Foley catheter was removed on postoperative day 1 or 2. An incentive spirometer was used throughout the hospital stay to promote full lung expansion and prevent respiratory complications. An early ambulation was encouraged. The patients would be instructed to get out of bed with assistance as much as they can tolerate it.

## RESULTS

Patient baseline data was shown as in Table 1. Among our 19 patients, 9 patients were men and 10 patients were women. Twelve patients had a level II ASA score, whereas 7 had a Level III. Median tumor size was 3.4 cm, (range: 1.3–6.2 cm). As for the tumor location, 2, 13, 4 tumors were located at upper, middle, and lower pole, respectively. The median diameter-axial-polar sum sore^16,17^ was 5 (range: 3–9). Zero-ischemic RAPN was successfully completed on 18 patients among our 19 study cases, as revealed in Table 2. Median operative time was 3.2 hours (range: 1.9–4.5 hours) and median blood loss was 100 cc (range: 30–950 cc). Intraoperatively, only 1 patient experienced severe bleeding (950 cc) and had to be converted to conventional open surgery. The median time to proper oral intake was 2 days (range: 1–21 days), and the median postoperative hospital stay was 4 days (range: 3–26 days). Only 1 patient developed postoperative complication. This male patient felt bloating, loss of appetite, dizziness, and tarry stools were found in the following days after surgery. After gastroscopy examination, stress ulcer over duodenum was confirmed, and he received proton pump inhibitor) treatment.

**TABLE 1:**
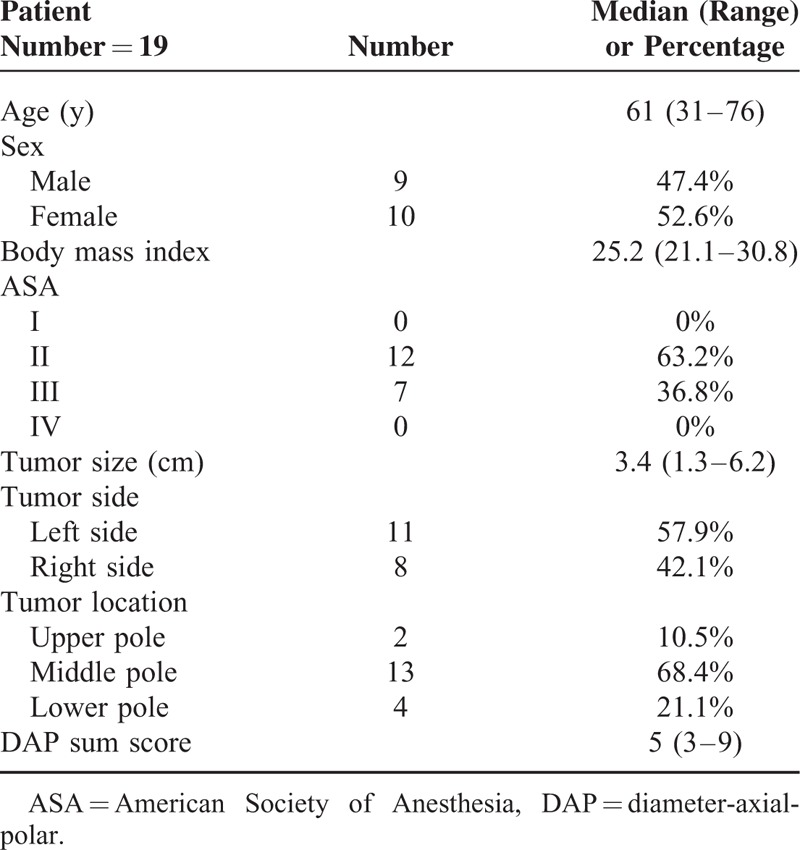
Base Line Data of Patients

**TABLE 2:**
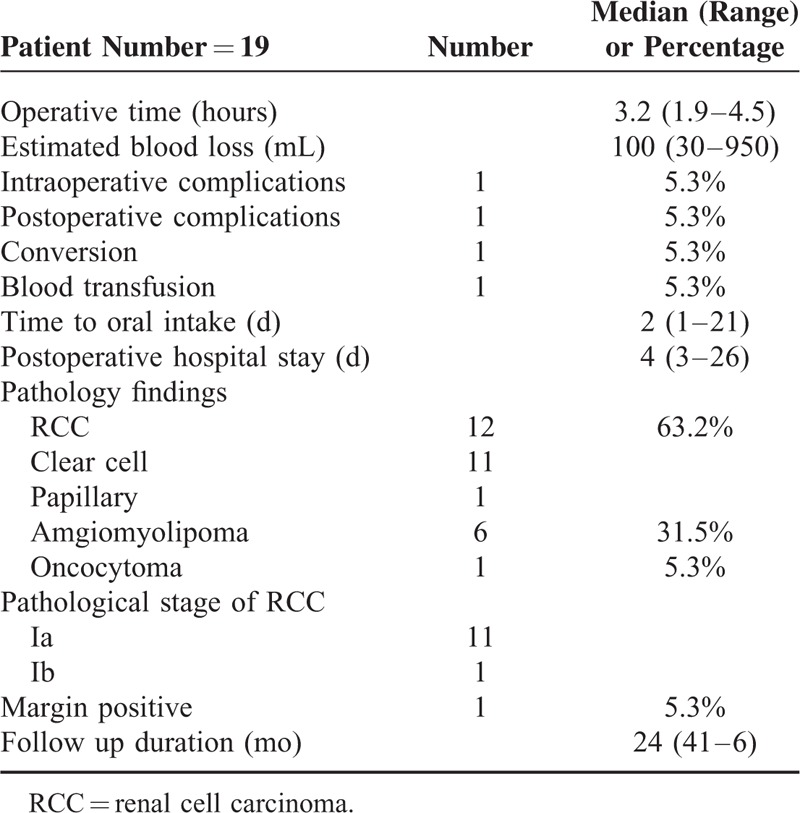
The Operative and Perioperative Data of the Patients

Histopathology confirmed renal cell carcinoma in 12 patients (63.2%, pathology stage Ia: 11, Ib: 1), angiomyolipoma in 6 patients, and oncocytoma in 1 patient. Surgical margin positive was found in 1 patient. Mean pre- and postoperative serum creatinine, eGFR, and Hb were comparable (Table 3). After being discharged from the hospital, our patient received either renal ultrasound or abdominal CT for follow up every 6 months. During the median observation duration of 24 months, neither tumor recurrence nor deterioration of renal function was detected among them.

**TABLE 3:**
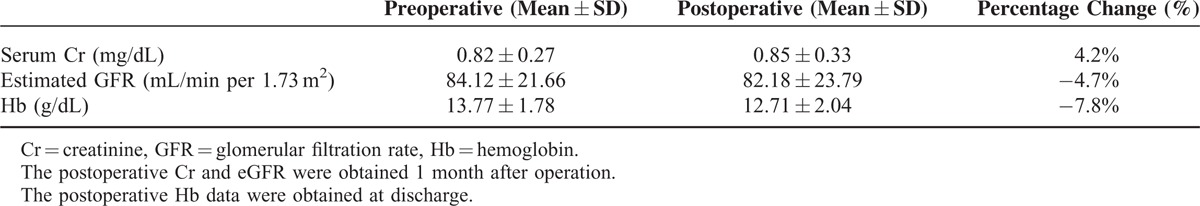
Renal Function and Hemoglobin Changes

## DISCUSSION

The study of Gill et al revealed that zero-ischemic partial nephrectomy can be performed via laparoscopy or robotic assistance,^13–15^ and the results were outstanding. We, however, believe that this operation is feasible but not reproducible, because excising the renal tumor by cold scissors without clamping the vessels was challenging and requires superb surgical technique. Herein, we proposed a novel and easy technique using a harmonic scalpel “drilling and clamping” method to implement zero-ischemic RAPN. The Harmonic scalpel is a surgical instrument used to simultaneously cut and cauterize tissue. The scalpel surface itself cuts through and cauterizes tissue by vibrating longitudinally at a 55,500 Hz rate.^18^ The high frequency vibration of tissue molecules generates stress and friction in tissue, which generates heat and causes protein denaturation.^18^ The range of peak tissue temperatures are much lower (60–100 °C) compared with those produced by electrocautery (200–300 °C), and as a result, the harmonic scalpel causes minimal energy transfer to surrounding tissue and potentially decreases the collateral damage.^19^ Furthermore, the harmonic scalpel is superior to traditional electrocautery in that it can cut through thicker tissue, creates less toxic surgical smoke, and may offer higher precision and less blood loss during operation.^19^ The Harmonic Scalpel has been widely used in laparoscopic procedures when hemostatic dissection with relatively little lateral thermal spread to neighboring tissue is desired, for example: cholecystectomy, hysterectomy, colorectal surgery, bariatric surgery, liver surgery, and splenectomy.^20–25^ The harmonic scalpel can also be installed on robotic arms to perform robotic-assisted hepatectomy,^26^ partial nephrectomy,^27^ and even cardiovascular surgery.^28,29^

The surrounding tissues of a kidney possessing a tumor are usually more hyper-vascular and replete with adhesions, especially the hilar region. Before clamping the vessels, whether clamping the main trunk or super-selectively controlling the tumor-specific renal arterial branches, one has to carefully distinguish the vessels from the adhesions. This process is not only time consuming and laborious, but also requires great skill and a considerably long learning curve. Unnecessary bleeding may also occur during this process. On the contrary, we have to neither approach the hilar region nor clearly separate the hilar vessels from adhesions when using the harmonic scalpel drilling and clamping methods. All we need to do is moderate mobilizing the kidney and separating it from the abdominal wall and psoas muscle, which is fairly easy to perform using robotic arms and hands. After identifying the tumor, we use the blunt blade of the harmonic scalpel to drill holes around the tumor, with a 5 mm distance from the tumor margin and 3 mm between adjacent holes. Then we insert the scalpel into the created holes, clamp, and then cut the tissue at power level 5 and 55,500 Hz vibrating frequency. The unique physical mechanism of a harmonic scalpel generates heat, causing protein denaturation, cutting the renal tissue with minimal bleeding. When excising the tumor by harmonic scalpel, electrocautery is unnecessary and peak tissue temperatures are reduced, leading to minimal energy transfer to surrounding renal parenchyma and potentially preserving more nephrone. After the tumor is removed, there is only slight oozing over the parenchymal resection defect. With the resulting clear view of the surgical site, we can easily suture the defect. Compared with excising the renal tumor by robotic cold scissors without vessel control, the harmonic scalpel drilling and clamping method is a safe, fast, and simple surgical technique. According to our data, the median operative time was 3.2 hours and median blood loss was 100 cc. Mean pre- and postoperative serum creatinine, eGFR, and Hb were comparable. During the median observation duration of 24 months, no tumor recurrence was detected. Our surgical results of this simple technique were noninferior to the report of Gill et al.^14^

There, however, were 2 patients whose treatment results were unsatisfactory, which revealed the limitations and disadvantages of this surgical method. The first patient had a 6.2 cm AML located on the upper pole and posterior aspect of the kidney. Although the tumor mass was exophytic in CT scan and seemed easy to identify, we found it very difficult to approach the target and separate this hyper vascular tumor from the adhesions, transperitoneally. Massive bleeding occurred when we tried to mobilize and rotate the kidney for a better operative space, and this patient was converted to conventional operation. The second patient had a 2.6 cm RCC, and the mass was also located at the upper pole and the posterior aspect of the kidney. The final pathology report showed a positive surgical margin. Unlike the robotic wrists, which have 360° flexibility, the harmonic scalpel installed on the robotic arm can only operate in a linear direction. As a result, when the tumor is located above and behind the kidney, it is very difficult to cut an arc and an unexpected bleeding or positive surgical margin may occur. Perhaps for such a tumor, a retroperitoneal approach may help the surgeon to obtain a better operation field. Furthermore, excising a tumor, which is too close to the hilum should not be approached using this surgical technique. Because the harmonic scalpel kerf is not as fine as that of robotic cold scissors, great vessel injury could occur.

## CONCLUSIONS

Zero-ischemic RAPN is now a subject of lively discussion and we believe that it will be a trend in the future. Our data revealed that a renal tumor, which is single, exophytic, and clinically T1, can be excised robotically by harmonic scalpel drilling and nonvascular clamping methods. This surgical technique is safe and effective procedure for renal sparing surgery. This future enabled a detail large number analysis of the safe and effective for renal tumor.
